# Energy-Harvesting Reinforcement Learning-based Offloading Decision Algorithm for Mobile Edge Computing Networks (EHRL)

**DOI:** 10.1371/journal.pone.0336903

**Published:** 2025-11-26

**Authors:** Hend Bayoumi, Nahla B. Abdel-Hamid, Amr M.T. Ali-Eldin, Labib M. Labib

**Affiliations:** Computers and Control Systems Engineering Department, Faculty of Engineering, Mansoura University, Mansoura, Egypt; Alma Mater Studiorum Universita di Bologna: Universita degli Studi di Bologna, ITALY

## Abstract

Mobile Edge Computing (MEC) is a computational paradigm that brings resources closer to the network edge to provide fast and efficient computing services for Mobile Devices (MDs). However, MDs are often constrained by limited energy and computational resources, which are insufficient to handle the high number of tasks. The problems of limited energy resources and the low computing capability of wireless nodes have led to the emergence of Wireless Power Transfer (WPT) and Energy Harvesting (EH) as a potential solution where electrical energy is transmitted wirelessly and then harvested by MDs and converted into power. This paper considers a wireless-powered MEC network employing a binary offloading policy, in which the computation tasks of MDs are either executed locally or fully offloaded to an edge server (ES). The objective is to optimize binary offloading decisions under dynamic wireless channel conditions and energy harvesting constraints. Hence, an Energy-Harvesting Reinforcement Learning-based Offloading Decision Algorithm (EHRL) is proposed. EHRL integrates Reinforcement Learning (RL) with Deep Neural Networks (DNNs) to dynamically optimize binary offloading decisions, which in turn obviates the requirement for manually labeled training data and thus avoids the need for solving complex optimization problems repeatedly. To enhance the offloading decision-making process, the algorithm incorporates the Newton-Raphson method for fast and efficient optimization of the computation rate under energy constraints. Simultaneously, the DNN is trained using the Nadam optimizer (Nesterov-accelerated Adaptive Moment Estimation), which combines the benefits of Adam and Nesterov momentum, offering improved convergence speed and training stability. The proposed algorithm addresses the dual challenges of limited energy availability in MDs and the need for efficient task offloading to minimize latency and maximize computational performance. Numerical results validate the superiority of the proposed approach, demonstrating significant gains in computation performance and time efficiency compared to conventional techniques, making real-time and optimal offloading design truly viable even in a fast-fading environment.

## 1. Introduction

The growing demand for applications that require response times and intensive computations fueled by technologies like Artificial Intelligence (AI) and the Internet of Things (IoT) has created a need for efficient computing with minimal delays. However, IoT devices and mobile gadgets often lack resources such as processing power, memory, and battery life [1]. Over the past two decades, Cloud Computing (CC) has enabled end-user devices to offload tasks to remote cloud data centers, providing access to remote computing and storage resources while reducing reliance on local processing, a process known as computation offloading [2,3]. While CC has traditionally addressed this challenge, it also comes with drawbacks like being far from user devices and consuming bandwidth and costs.

To overcome these difficulties, MEC has emerged as an approach to bring computing resources closer to end devices [4]. MEC enables mobile devices to perform computational offloading by wirelessly transmitting their operations and data to the edge layer, thus minimizing the delays introduced by CC. This enables tasks to be transferred to the edge layer through communication, resulting in reduced data transmission time and device response time, reduced pressure on network bandwidth, reduced energy consumed, reduced cost of data transmission, and also achieved decentralization [5].

In MEC, offloading can occur in two scenarios: binary offloading, where all the tasks are either computed locally or offloaded to the edge server, and partial offloading, where part of the data is offloaded to the edge server and the remaining part is computed locally [6]. Offloading tasks in MEC can improve application performance. However, the growing number of devices has introduced new issues such as network congestion and resource allocation problems [7]. Also, offloading may not be a suitable solution for delay-sensitive applications. Therefore, it is crucial to optimize offloading decisions to address these issues.

Using deep learning (DL) strategies to learn future directions from data allows us to solve these challenges by making complex offloading decisions more efficient [8]. But for truly demanding applications, with stringent latency requirements, even optimal offloading might falter. This is where reinforcement learning steps in. RL is one of the essential types of DL where an agent learns how to make the best decision based on experiences gained from the environment [9].

On the other hand, while MEC plays a role in improving the performance of applications that require computing and low latency, the limited battery capacity of devices creates an energy bottleneck that impacts network performance [10]. The development of wireless Energy Harvesting (EH) technologies, such as renewable EH and WPT, offers a sustainable solution for replenishing energy-constrained MDs by harvesting energy transmitted from a centralized ES [11]. This paper explores the development of an online offloading algorithm for a wireless-powered MEC network, consisting of a single ES and multiple MDs. Each MD operates under a binary offloading policy. The primary objective is to optimize the network’s computation rate which represents the number of processed bits within a unit of time while minimizing the loss which refers to reducing the error between the predicted and actual optimal offloading decisions during training, and reducing the computation time which involves both minimizing the total latency experienced by mobile devices and accelerating the optimization process itself during algorithm training.

To achieve this goal, the Energy-Harvesting Reinforcement Learning-based Offloading Decision Algorithm EHRL is proposed. Unlike existing MEC–WPT studies that rely on conventional RL algorithms or gradient-based optimization methods, our work uniquely integrates the Newton–Raphson method with the Nadam optimizer within an RL–DNN framework for faster convergence, reduced overall computation time, and training loss. The evaluation of the EHRL algorithm shows that it maximizes the weighted sum computation rate of all the MDs, while significantly reducing the total network time by more than an order of magnitude. Such a performance improvement enables the practical implementation of real-time and optimal designs in wireless-powered MEC networks, even in fast-fading environments.

The organization of the paper is as follows. Section 2 begins with a comprehensive review of the literature. In Section 3, the methodology behind the proposed algorithm is discussed. The proposed algorithm is thoroughly explained in Section 4. Section 5 is dedicated to the presentation of numerical results. The paper is then concluded in Section 6.

## 2. Related work

This section provides an overview of the research on computational offloading for MEC, using WPT and RL-based approaches to facilitate offloading.

Bi et al. [12] studied maximizing the weighted sum computation rate in multi-user, wireless-powered edge computing networks with binary computation offloading policies. They proposed two efficient solution algorithms to mitigate the complicated combinatorial mode selection problem via a partitioned optimization framework wherein mode selection is treated as fixed a priori. The authors suggested a simple bisection search method to compute the conditionally optimal time allocation, and then constructed the algorithm to apply the coordinate descent on the selected mode. An alternating direction method of multipliers (ADMM) method was proposed to optimize them jointly. In [13], Huang et al. investigated a wireless-powered MEC network employing binary offloading. They propose a Deep Reinforcement learning-based Online Offloading (DROO) framework that utilizes a deep neural network (DNN) to learn binary offloading decisions from experience, improving the produced offloading and reducing computational complexity actions as it does not necessitate any manually labeled training data, especially in large-scale networks. An order-preserving quantization and an adaptive parameter setting method were also devised to achieve fast algorithm convergence.

A Lyapunov-guided deep reinforcement learning (RL) approach was studied in [14] and focused on developing a stable online computation offloading framework. This framework accounts for random task arrivals, which optimizes energy consumption while stabilizing data queuing. The study’s optimization problem involved binary offloading decisions and system resource allocation, which was solved through a combination of Lyapunov optimization and deep learning. The proposed framework was shown to efficiently compute optimal decisions for offloading tasks and reallocating system resources in very short processing times. In [15], Zhang et al. developed an online framework based on Deep Reinforcement Learning (DRL) and proposed a DRL-based algorithm to achieve the near-maximal computation rate where a DNN, equipped with specific exploration and training strategies, is employed to determine the near-optimal WPT duration. Additionally, an efficient algorithm is devised to solve a related sub-problem. Mustafa et al. [16] proposed a reinforcement learning-based intelligent online offloading (RLIO) framework for optimizing task offloading in complex network scenarios. The framework dynamically decides between local and remote computation to maximize performance under varying wireless channel conditions.

The paper [17] introduces a novel approach to addressing the challenge of optimal offloading decision-making in the context of power resource constraints. It proposes a deep reinforcement learning (DRL) algorithm to optimize the offloading decision while considering limited computational resources and the trade-off between latency and energy consumption. The algorithm decouples the original problem into a top problem of optimizing binary offloading decisions and a sub-problem of optimizing transmitted powers and WPT duration under a given offloading decision. Subsequently, a self-learning DRL framework is designed to output near-optimal offloading decisions, thereby maximizing resource allocation efficiency. Considering the computation rate maximization problem in a WPT-empowered MEC with multiple WDs and multiple HAPs, Zhang et al. [18] proposed a DRL-based algorithm to output the near-optimal offloading decision and designed an efficient algorithm based on the Lagrangian duality method to efficiently derive the optimal time allocation strategy. A novel deep reinforcement learning framework was proposed in [19] to address the challenge of optimizing offloading strategies and WPT duration jointly. This framework leverages orthogonal frequency division multiple access (OFDMA) for channel access and tackles the resulting non-convex problem by decomposing it into two sub-problems: determining the most effective offloading solution and allocating optimal WPT time. This approach aims to identify a synergistic solution that maximizes system performance. A DRL-based Adaptive Offloading algorithm for WPT-MEC, referred to as the DRL-Based Adaptive Offloading (DRLAO) algorithm, was proposed in [20]. DRLAO is designed to dynamically adapt to fluctuating environmental conditions, make swift decisions, and adjust parameters in real time. It includes an Augmented Deep Neural Network (AugDNN) to learn optimal strategies, Order-Preserving Quantization (KOQ) to promote the offloading of decision-making, and a Modified Secant Method (MSM) for optimizing electrical energy allocation. The following Table 1 summarizes the related work.

**Table 1:** Related Work Summary.

Ref	Methodology	Pros	Cons
Suhzi B. et al. [12] 2018	Adopts a binary computation offloading policy, the problem is formulated as a joint optimization of computing mode selection and transmission time allocation. Proposing two optimization methods: • Decoupled optimization (bi-section search and coordinate descent). • Alternating Direction Method of Multipliers (ADMM)-based joint optimization (breaks a complex problem into smaller sub-problems to be solved independently)	ADMM-based optimization offers low complexity and scales well with increasing network size. The study provides valuable insights into computing mode selection, giving priority to stronger wireless channels over weaker ones.	The coordinate descent method can suffer from high computational complexity in large networks.
Liang Huang et al. [13] 2020	A Deep Reinforcement Learning-based Online Offloading (DROO) framework, which uses a DNN to make offloading decisions. The optimization problem is solved using a bisection search. DROO does not require labeled training data as it continuously improves its policy using reinforcement learning.	DROO reduces computation time compared to existing optimization algorithms. Ensures adaptability as it automatically adjusts its parameters in response to any network changes. Offers scalability by avoiding the “curse of dimensionality” often encountered in traditional RL.	Bi-section search follows a logarithmic convergence rate, meaning it requires multiple iterations to reach a highly accurate solution, leading to slow convergence.
Suhzi B. et al. [14] 2021	LyDROO (Lyapunov-guided Deep Reinforcement Learning-based Online Offloading) is proposed, combining: • Lyapunov optimization (to ensure long-term queue stability and power constraints). • DRL (to make real-time offloading decisions using an actor-critic DNN model).	LyDROO reduces the computational complexity compared to conventional MINLP solvers. The combination of Lyapunov optimization and DRL results in faster convergence.	Lyapunov-based methods often require solving optimization problems within a time frame, which can be costly in large networks.
Shubin Zhang et al. [15] 2022	Introduces partial offloading, where tasks are split between local computations and offloading. Involves a DRL-based approach to learn the near-optimal WPT duration. Involves the Lagrangian duality method to solve the subproblem of optimizing offloading and energy allocation.	Supports partial offloading, making it more flexible than traditional binary offloading models Can dynamically adjust offloading decisions based on real-time channel variations and energy constraints.	The combination of DRL and Lagrangian duality still requires significant processing power, which may not be feasible for all devices. Since local computing and offloading consume energy, improper parameter tuning could lead to inefficient energy usage.
Ehzaz Mustafa et al. [16] 2023	A Reinforcement Learning-based Intelligent Online Offloading (RLIO) framework, which makes offloading decisions in real-time based on: • Dynamic wireless channel conditions. • A convolutional neural network (CNN) • RL is used to train the model to evaluate the best offloading decisions over time.	Real-time decision-making, since it quickly responds to changing wireless conditions, Scalability, since the CNN-based RLIO framework quickly responds to changing wireless conditions**,** making it suitable for dense networks.	The CNN-based approach still has higher processing requirements and requires a training phase that increases training overhead.
Guanqun Shen et al. [17] 2023	A wireless-powered mobile edge computing (WP-MEC) network using binary offloading based on: • A deep neural network (DNN) is used to generate near-optimal offloading decisions. • The optimization problem is formulated as a Mixed Integer Nonlinear Programming (MINLP) problem. • The resource allocation problem is solved using Lagrangian dual theory, providing a closed-form optimal solution.	The framework dynamically adjusts offloading decisions based on real-time changes in channel conditions and energy levels. The algorithm is suitable for large-scale MEC networks.	The DRL framework requires an initial learning phase, which may introduce a delay before achieving optimal performance and increase the overhead.
Shubin Zhang et al. [18] 2024	The paper considers a wireless-powered mobile edge computing (WP-MEC) network with multiple hybrid access points (HAPs) and jointly optimizing: • Offloading decisions • Time allocation for WPT and task offloading. • Time allocation using a DRL-based algorithm and a Lagrangian duality-based optimization method.	Compared to traditional actor-critic (AC) algorithms, the proposed DRL framework converges faster and achieves higher computation rates. The model can dynamically adjust to varying wireless conditions, making it effective for large-scale IoT networks.	The combination of DRL and Lagrangian duality still demands high processing power, which may not be ideal for low-power IoT devices and might increase the training overhead.
Mohammed Maray et al. [19] 2024	The optimization problem is formulated as a joint DRL-based framework, where: • A Convolutional Neural Network (CNN) is used to generate optimal binary offloading decisions. • Orthogonal Frequency Division Multiple Access (OFDMA) is used for communication to minimize interference. • A one-dimensional search method optimizes the WPT duration for maximum computation efficiency.	The framework handles large-scale dynamic networks, achieving low execution latency. The use of CNN for offloading decisions and a single-parameter optimization approach simplifies computation.	Limited applicability: The framework assumes WPT availability, which may not be feasible in all scenarios.
Xiaojun Wu et al. [20] 2024	A Deep Reinforcement Learning-Based Adaptive Offloading (DRLAO) framework designed to Optimize binary offloading decisions dynamically and Allocate power resources efficiently. It consists of three key components: • Augmented Deep Neural Network (AugDNN): to Learn from past experiences. • Order-Preserving Quantization (KOQ): to obtain binary choices. • Modified Secant Method (MSM): Solve the energy allocation sub-problem efficiently.	Faster convergence as it reduces CPU execution latency and minimizes oscillations in changing environments. Automatically adjusts offloading and energy allocation without human intervention.	Although optimized, the combination of DNN, KOQ, and MSM still demands significant processing power leading to Computational complexity.

## 3. Methodology

**Fig 1:**
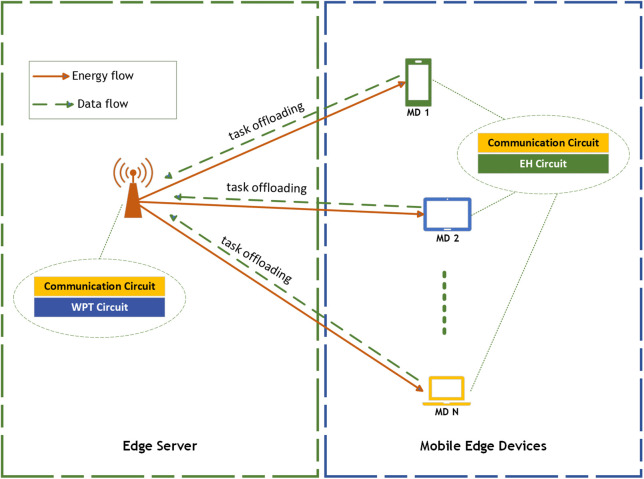
An Example of a WPT-MEC Network.

A wireless power transfer WPT-MEC network is considered, as shown in Fig 1. This network consists of an ES device and a number N of edge MDs. The ES has a stable power supply, and it is presumed to possess higher computation capability than the MDs. This is because the ES receives the offloaded computation tasks from the MDs, and it can also wirelessly distribute energy to them using Radio Frequency (RF) signals. Each MD is equipped with a rechargeable battery that can store the harvested energy to power the upcoming operations in the network. The total time T represents the complete duration required for an offloading process, from energy harvesting to task execution and result transmission. It can be decomposed into several key components: Energy Harvesting Time, Task Offloading Time, Computation Time, and Transmission/download Time.

### 3.1. Energy harvesting

The MDs received RF energy radiated by the ES. To harvest the received energy from ES, an MD takes time βT, β represents the fraction of the total time frame T that a MD dedicates to harvesting energy from the ES, β ∈ [0, 1]. The amount of energy harvested depends on the power of the RF signals, the efficiency of the energy harvesting circuit, and the time spent harvesting. The harvested energy can be expressed as [21]:


$$E_{i}=\mu Pg_{i}\beta T$$ (1)

where $g_{i}$ Denotes the wireless channel gain between the ES and the i-th MD, P is the ES transmit power, and µ ∈ (0, 1) is the energy harvesting efficiency.

### 3.2. Computational modes

The binary task offloading policy is widely adopted for handling non-partitionable, simple sensing tasks in IoT networks [6], where the task is either executed locally on the edge device or entirely offloaded to the edge server. Let $f_{i}$ ∈ {0,1} be an indicator variable. If $f_{i}$ = 1, the computation task of the i-th MD is offloaded to the ES, whereas if $f_{i}$= 0, the task is executed locally on the device.

a. **Local computing:**

In the local computing mode, a MD has the capability to both harvest energy and perform its computing tasks simultaneously [22]. The amount of processed bits by the MD can be calculated as $S_{i}t_{i}/c$, where $S_{i}$ represents the processor’s computing speed (cycles per second), $t_{i}$ represents the computation time, and c denotes the number of CPU cycles required to process a single bit of task data [23]. The energy that MD consumes for computing can be computed by $k_{i}S_{i}^{\text{3}}t_{i}\leq E_{i}$, where $k_{i}$ represents the energy efficiency coefficient related to computation for thr i-th device [24]. To maximize the amount of data processed within a given time T, the harvested energy will be depleted, and $S_{i}$ can be calculated as $S_{i}^{*}={\left(E_{i}/k_{i}T\right)}^{\frac{\text{1}}{\text{3}}}$ [23]. Hence, the computation rate x in this case, considering Equation (1) along with other relevant factors, can be determined as:


$$x_{L,i}^{*}(\beta )=\frac{S_{i}^{*}t_{i}^{*}}{cT}=\phi_{\text{1}}{\left(\frac{g_{i}}{k_{i}}\right)}^{\frac{\text{1}}{\text{3}}}\beta^{\frac{\text{1}}{\text{3}}},$$ (2)

where $\phi ≜(\mu P{)}^{\frac{\text{1}}{\text{3}}}/c$ is a fixed parameter, L for local.

b. **Computational offloading:**

The i-th MD’s offloading time is represented as $\in_{i}T$, where $\in_{i}$ is the offloading ratio and $\in_{i}$ ∈ [0, 1]. Here, the ES’s transmission power and processing speed are assumed to be far greater than those of the MDs. Also, compared to the data offloaded to the edge server, the computation result that needs to be downloaded to the MD is substantially shorter. As a result, the time that ES takes to compute and download tasks can be safely neglected, meaning that the MDs just need to expend energy and time on offloading data [25]. In task offloading, an MD exhausts all its harvested energy, so the computation rate can be maximized $P_{i}^{*}=E_{i}\in_{i}T$. Therefore, the computation rate is equivalent to the data offloading capacity as expressed in Equation (3) [22,25]:


$$x_{O,i}^{*}\left(\beta ,\in_{i}\right)=\frac{B\in_{i}}{v_{u}}{\text{log}}_{\text{2}}\left(\text{1}+\frac{\mu P\beta g_{i}^{\text{2}}}{\in_{i}N_{\text{0}}}\right)$$ (3)

where B is the channel bandwidth, $v_{u}$ denotes edge computing overhead, $N_{\text{0}}$ denotes the ES’s receiver noise power, O for Offloading. For clarity and ease of reference, the key symbols used throughout this paper are summarized in Table 2.

**Table 2:** Summary of Notations Used in this paper.

Symbol	Description
N	Number of mobile devices
$g_{i}$	Channel gain for the node i
μ	Energy Harvesting Efficiency
T	Total Time
P	Transmitted Power
$E_{i}$	Harvested Energy at the node i
$f_{i}$	Offloading indicator where $f_{i}=\text{1},taskoffloaded$ $f_{i}=\text{0},executedlocaly$
c	CPU cycles required per bit
$k_{i}$	Energy efficiency coefficient
β	Fraction of time for harvesting energy
$\in_{i}$	Offloading ratio
B	Channel Bandwidth
$v_{u}$	Edge computing overhead
$N_{0}$	Noise Power
$w_{i}$	Weight assigned to node i

### 3.3. Problem formulation

Based on Equations (2) and (3), the weighted sum computation rate of the wireless-powered MEC network within a given time frame can be expressed as:


$$Q(g,\text{f},\in ,\beta )≜\sum_{i=\text{1}}^{N}\;w_{i}\left(\left(\text{1}-f_{i}\right)x_{L,i}^{*}(\beta )+f_{i}x_{O,i}^{*}\left(\beta ,\in_{i}\right)\right)$$ (4)

where ${\text{w}}_{\text{i}}$ is the weight assigned to the i-th MD.

In the context of (4), the computation rate of the MEC network depends on the offloading decisions and the transmitted power within each specific time frame characterized by channel gain g. The primary objective is to maximize the weighted sum computation rate:


$$\begin{array}{cc} (P):Q^{*}(g)=\underset{f,\in ,\beta }{\text{maximize}} & Q(g,f,\in ,\beta )\\ \text{subject to} & \sum_{i=\text{1}}^{N}\;\in_{i}+\beta \leq \text{1},\\ & \beta \geq \text{0},\in_{i}\geq \text{0},\forall i\in N,\\ & f_{i}\in \{\text{0},\text{1}\}. \end{array}$$ (5)

Since f is given, the problem (P) can be given as follows:


$$v^{\left(t+\text{1}\right)}=v^{\left(t\right)}-\frac{Q\left(v^{\left(t\right)}\right)}{dQ\left(v^{\left(t\right)}\right)}$$ (6)

The problem (Pis solved to compute the corresponding computation rate $Q^{*}(g,f)$. This requires finding the optimal energy harvesting and offloading time allocations (∈,β) that maximize the weighted sum computation rate under fixed offloading decisions. Since this optimization problem is nonlinear and constrained, the Newton-Raphson method was adopted to efficiently find the optimal values of (∈,β). Specifically, the computation rate is modeled as a differentiable function Q(v) of an optimization variable v∈{∈i,β} and its gradient is computed as dQ(v). The value v is iteratively updated as follows [26]:


$$v^{\left(t+\text{1}\right)}=v^{\left(t\right)}-\frac{Q\left(v^{\left(t\right)}\right)}{dQ\left(v^{\left(t\right)}\right)}$$ (7)

The update is repeated until the change between iterations is less than a specified tolerance or until a maximum number of iterations is reached. A Pseudo-code for the Newton-Raphson method algorithm is provided in Algorithm 1. 
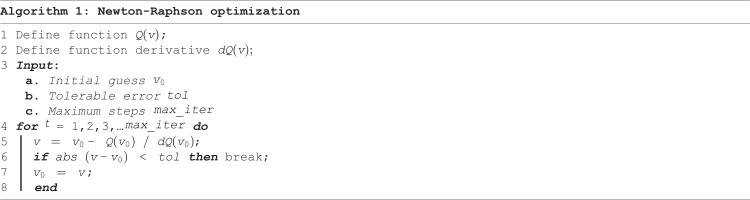


## 4. Proposed algorithm

**Fig 2:**
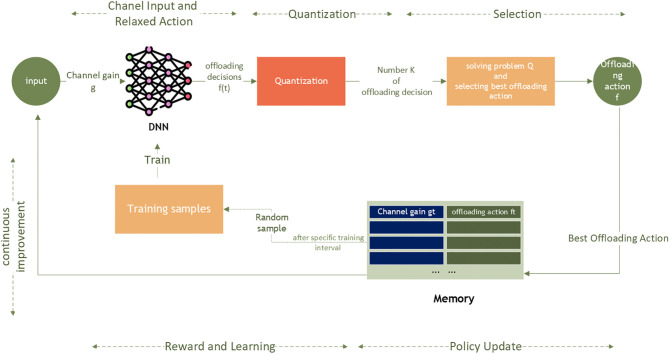
Proposed Algorithm.

The algorithm’s framework is depicted in Fig 2, where the creation of offloading actions hinges on employing a DNN defined by its embedded parameters θ, which are the weights linking the hidden neurons. The proposed algorithm is composed of 6 stages and can be summarized as follows:

1. **Channel Input and Relaxed Action:** Utilizing the current channel gain g(t) as its input, the DNN forecasts a relaxed offloading decision $f_{t}$ according to its existing offloading policy. The main objective of the offloading function π is to identify the optimal offloading decision $f_{t}$ upon revelation of the channel state at the beginning of each time framet. This offloading policy can be derived as:

$$\pi :g\to f^{*}$$ (8)

2. **Quantization**: The relaxed action ${\hat{f}}_{t}$ is transformed into K binary options, where 1 signifies offloading and 0 indicates local computing. This quantization process utilizes an order-preserving quantization method [13] that limits the output to a maximum of N binary offloading actions, corresponding to the number of MDs involved. Subsequently, the optimal action $f_{t}$ is chosen based on the computation rate achievable, as outlined in (P).
3. **Selection:** Using the Newton-Raphson method [26], the computation rate is determined. Subsequently, the network analyzes the achievable computation rate for each option and chooses the optimal action $f_{t}$ according to:

$$f_{t}^{*}=arg\underset{f_{i}\in \left\{f_{k}\right\}}{max}\;Q^{*}\left(g_{t},f_{i}\right)$$ (9)

4. **Reward and Learning:** The network receives a reward $Q(g_{t},f_{t}$and the acquired optimal offloading action will be utilized to refine and update the offloading policy of the DNN. Accordingly, at time frame t, the pair $\left(g_{t},f_{t}\right)$ is added to the memory as a new training sample for future use. Initially, the memory has a restricted capacity and as it becomes full, the most recent data sample replaces the oldest one.
5. **Policy Update:** Unlike existing DNN approaches, which are based on supervised learning where a large number of manually labeled training samples are required, and by adapting the offloading decision policy automatically when the channel distribution varies, the proposed method removes the necessity for manually generated samples and is therefore more appropriate for dynamic wireless applications. Periodically, the system samples past experiences from memory and uses them to train the DNN. This training refines the DNN’s parameters for better decision-making. The parameters $\theta_{t}$ of The DNN are updated by applying the Nadam algorithm as in (10) [27] to reduce the averaged cross-entropy loss, as shown in (11):

$$\theta_{t+\text{1}}=\theta_{t}-\frac{\eta }{\sqrt{{\hat{v}}_{t}}+\in }\left(\beta_{\text{1}}{\hat{m}}_{t}+\frac{\left(\text{1}-\beta_{t}\right)g_{t}}{\text{1}-\beta_{\text{1}}^{t}}\right)$$ (10)

$$L\left(\theta_{t}\right)=-\frac{\text{1}}{\left|T_{t}\right|}\sum_{\in \in T_{t}}\;\left({\left({𝐱}_{\in }^{*}\right)}^{⊤}\text{log}f_{\theta_{t}}\left({𝐠}_{\in }\right)+{\left(\text{1}-{𝐟}_{\in }^{*}\right)}^{⊤}\text{log}\left(\text{1}-f_{\theta_{t}}\left({𝐠}_{\in }\right)\right)\right)$$ (11)

where ${\hat{m}}_{t},{\hat{v}}_{t}$ are the first and Second moment estimates, respectively. $\left|T_{t}\right|$Indicates the size of the set $T_{t}$, the superscript ⊤ represents the transpose operator and the logarithmic function refers to the element-wise logarithm applied to a vector. 
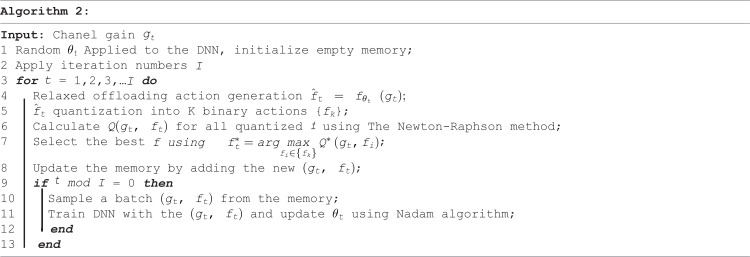

6. **Continuous Improvement:** With each new channel gain $(g_{t+\text{1}}),$ the DNN generates a new optimal offloading decision $\left(f_{t+\text{1}}\right)$ using its updated parameters. The reinforcement learning process is repeated as new channel realizations are observed, allowing the DNN to continuously refine its policy and offloading strategies over a predefined number of time frames. Additionally, due to the limited memory space available, DNN focuses on learning from the latest high-quality data samples. A Pseudo-code for the proposed algorithm is provided in Algorithm 2.

## 5. Results

### 5.1. Dataset

The used dataset provides time-varying small-scale fading coefficients for multiple users under realistic wireless propagation assumptions, including path-loss, fading, and shadowing effects [13]. N MDs are randomly located in an area of 100 X 100 m with one ES. The wireless channel between each MD and the ES is modeled using a standard log-distance path-loss model with reference loss $PL(d_{\text{0}})=-\text{3}\text{0}dB$ at $d_{\text{0}}=\text{1}m$ and path-loss exponent α=3.5. Small-scale fading follows a Rayleigh distribution, Large-scale shadowing is modeled as a log-normal random variable with standard deviation σ=6dB. The RF-to-DC energy harvesting efficiency is set to μ = 0.7. The noise power spectral density is fixed at $N_{\text{0}}={\text{1}\text{0}}^{-\text{1}\text{0}}W/Hz$, and the system bandwidth is B=2MHz.

### 5.2. Simulation setup

The performance of the proposed algorithm is assessed in this section, focusing on the computation rate metric. In the simulations, we use the channel gain matrix with N = 10 wireless devices over n = 3000 time frames. DNN in the proposed work comprises an input layer, two hidden layers, and an output layer. The initial hidden layer is equipped with 120 neurons, while the subsequent hidden layer has 80 neurons. Within the domain of neural networks, the well-established universal approximation theorem posits that a single hidden layer with a significant number of neurons can effectively approximate any continuous function f, provided suitable activation functions like sigmoid, ReLU, and tanh are utilized [28]. In this algorithm, ReLU acts as the activation function for the hidden layers, and the sigmoid function is used in the output layer for data processing. Reinforcement learning parameters include learning rate 0.001 (Nadam optimizer), replay memory capacity of 1024 transitions, and batch size of 128. The fixed task size per offloading decision is $\text{5}x{\text{1}\text{0}}^{\text{6}}$ bits, which is consistent with benchmark MEC scenarios. Simulations were executed using a laptop configuration with an Intel Core i7-10510U 2.3 GHz CPU and 16 GB RAM. The algorithm is implemented in Python with TensorFlow 2.0 and the simulation parameters are set as provided in Table 3.

**Table 3:** Simulation parameters.

Parameter	value
Reference path-loss at 1 m ${𝐏𝐋}_{0}$	−30 dB
Path-loss exponent α	3.5
Shadowing variance σ	6 dB
Fading distribution	Rayleigh
Transmitting power of ES P	3 W
Time	1 s
Energy harvesting efficiency μ	0.7
Channel bandwidth B	2 x ${\text{1}\text{0}}^{\text{6}}$ Hz
Noise power $N_{0}$	${\text{1}\text{0}}^{-\text{1}\text{0}}$ w
**Computation cycle** c ** to calculate one-bit data**	100 Cycles/bit
Computation energy efficiency coefficient $k_{i}$ [29]	${\text{1}\text{0}}^{-\text{2}\text{6}}$ J/cycle
edge computing overhead $v_{u}$	1.1
RL optimizer	NADAM
Memory size	1024 Bytes
Training batch size	128
Nadam learning rate	0.001
Task size	5x ${\text{1}\text{0}}^{\text{6}}$ bits

When addressing the challenge of computing the weighted sum in problem (P), we use Newton’s Raphson search method, which is generally faster and more accurate compared to other methods [26].

**Fig 3:**
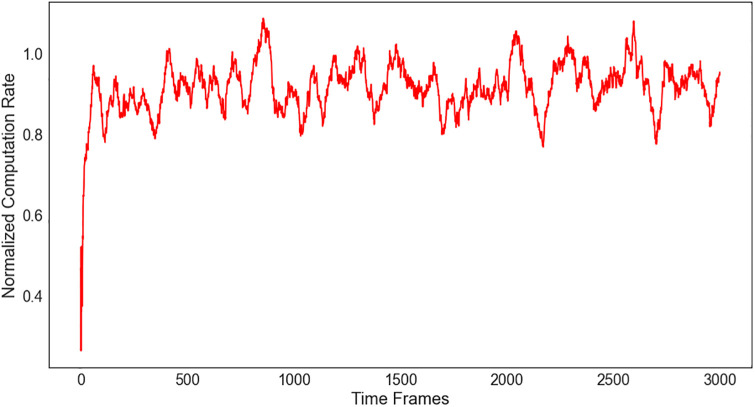
Normalized computation rate for N = 10.

Fig 3 shows a plot of the normalized computation rate Q∘, as calculated in (12), where l is set to be fixed and K=N. We can see that the moving average Q∘ of EHRL gradually converges to the optimal solution when t is large. Regarding sample complexity, the proposed method reaches over 95% of its final normalized computation rate within approximately the first 200 time frames, as observed empirically. This fast convergence is attributed to (i) the quadratic convergence property of Newton–Raphson in optimizing the continuous resource allocation, and (ii) the Nadam optimizer accelerating the DNN parameter updates. Thus, our approach offers both rapid convergence and low per-iteration complexity, making it practical for real-time mobile edge computing scenarios.

**Fig 4:**
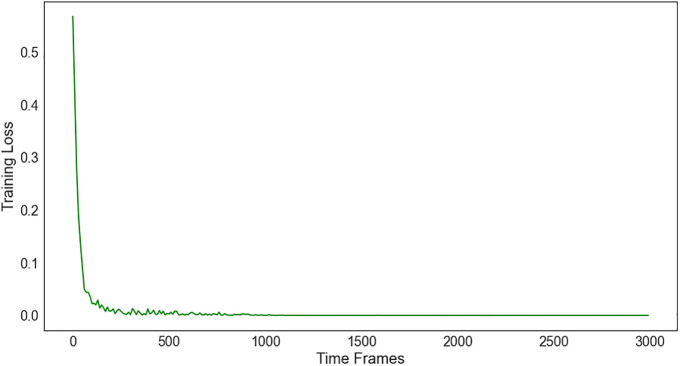
Training losses for N = 10.


As shown in Fig 4, the training loss L(θt) gradually decreases and eventually settles at a value close to 0. This stability is punctuated by the occasional random fluctuations, which arise mainly from the random sampling of training data.

$$\hat{Q}(\text{g},\text{f})=\frac{Q^{*}(\text{g},\text{f})}{\underset{{\text{f}}^{\prime }\in \{\text{0},\text{1}{\}}^{N}}{max}\;Q^{*}\left(\text{g},{\text{f}}^{\prime }\right)},$$ (12)

**Fig 5:**
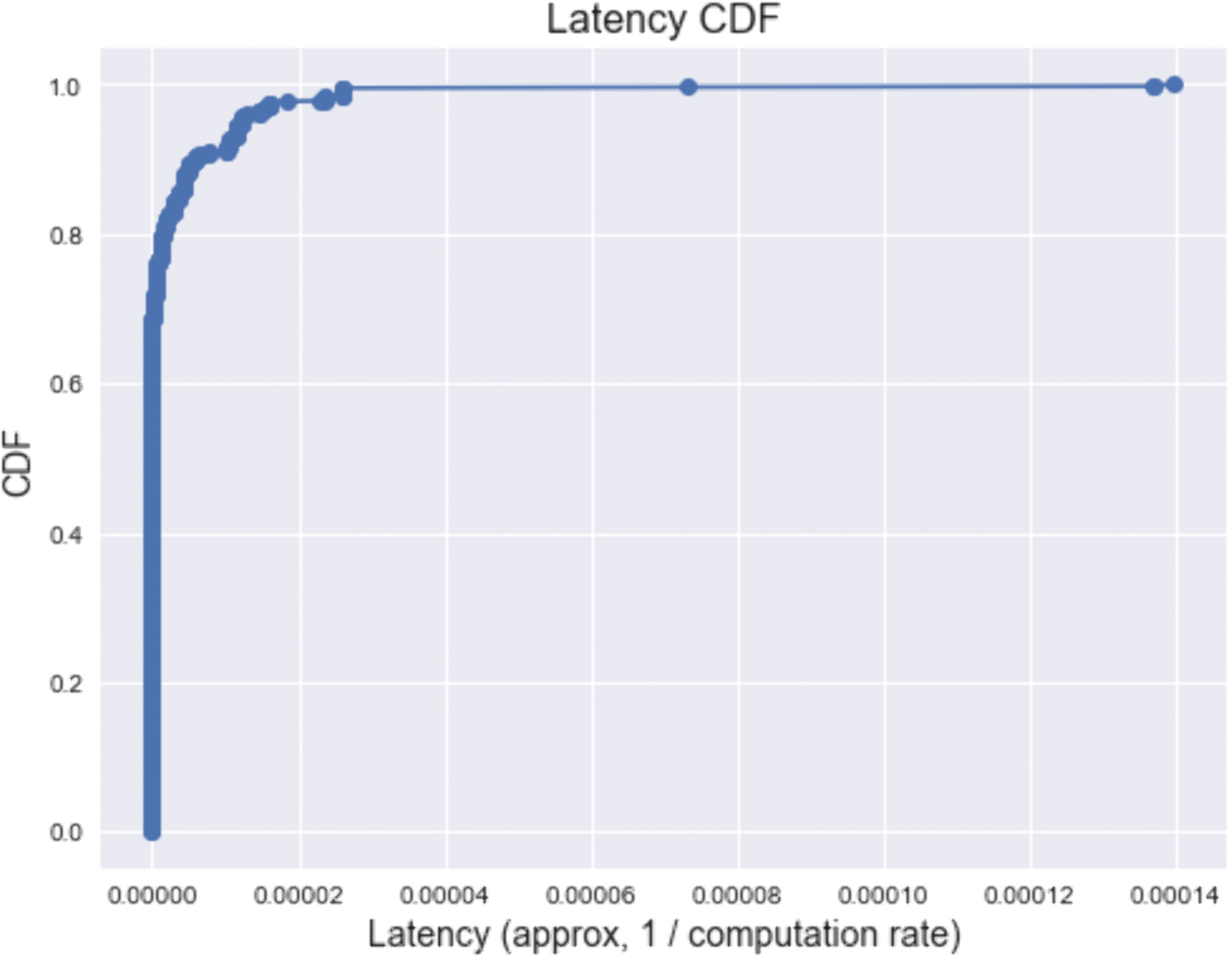
CDF of task latency, approximated as the inverse of the achieved computation rate.

Fig 5 illustrates the Cumulative Distribution Function (CDF) of task latency, where latency is approximated as the inverse of the achieved computation rate. The curve rises sharply and reaches a CDF value of 1.0 within a latency range of 0–8 × 10 − 14 seconds, indicating that nearly all tasks are completed with extremely low delay. This steep slope reflects the high responsiveness of the proposed Newton–Raphson–enhanced Nadam optimization framework, which ensures rapid convergence toward optimal offloading decisions and **r**eduction in total execution time, confirming the method’s capability to minimize latency while maintaining high computation rates.

**Fig 6:**
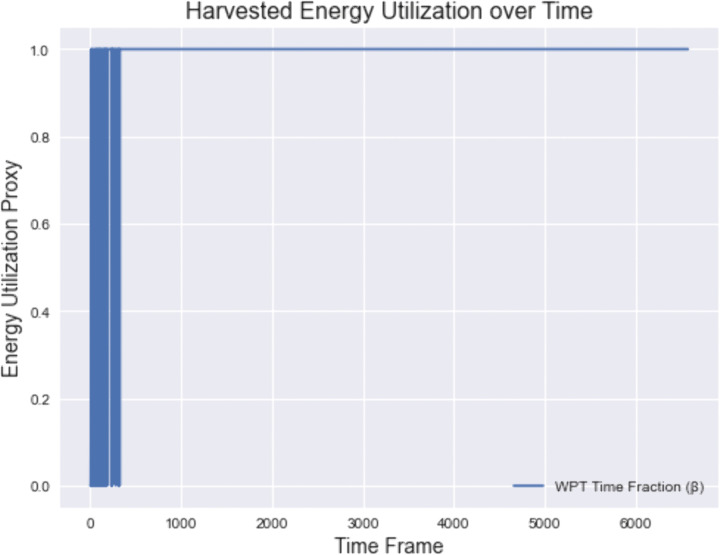
The evolution of the Wireless Power Transfer (WPT) time fraction β over simulation frames.

Fig 6 presents the evolution of the harvested energy utilization over time, quantified via the WPT time fraction β. During the initial frames, β exhibits small fluctuations due to the exploration phase of the RL policy. As training progresses, β converges to approximately 1.0, indicating that the system consistently allocates the maximum available WPT time for energy harvesting. This high and stable utilization ensures that mobile devices operate with sufficient energy for computation tasks, complementing the latency results in Fig 5. Together, these findings confirm that the proposed approach effectively balances energy harvesting efficiency and low-latency task execution.

**Fig 7:**
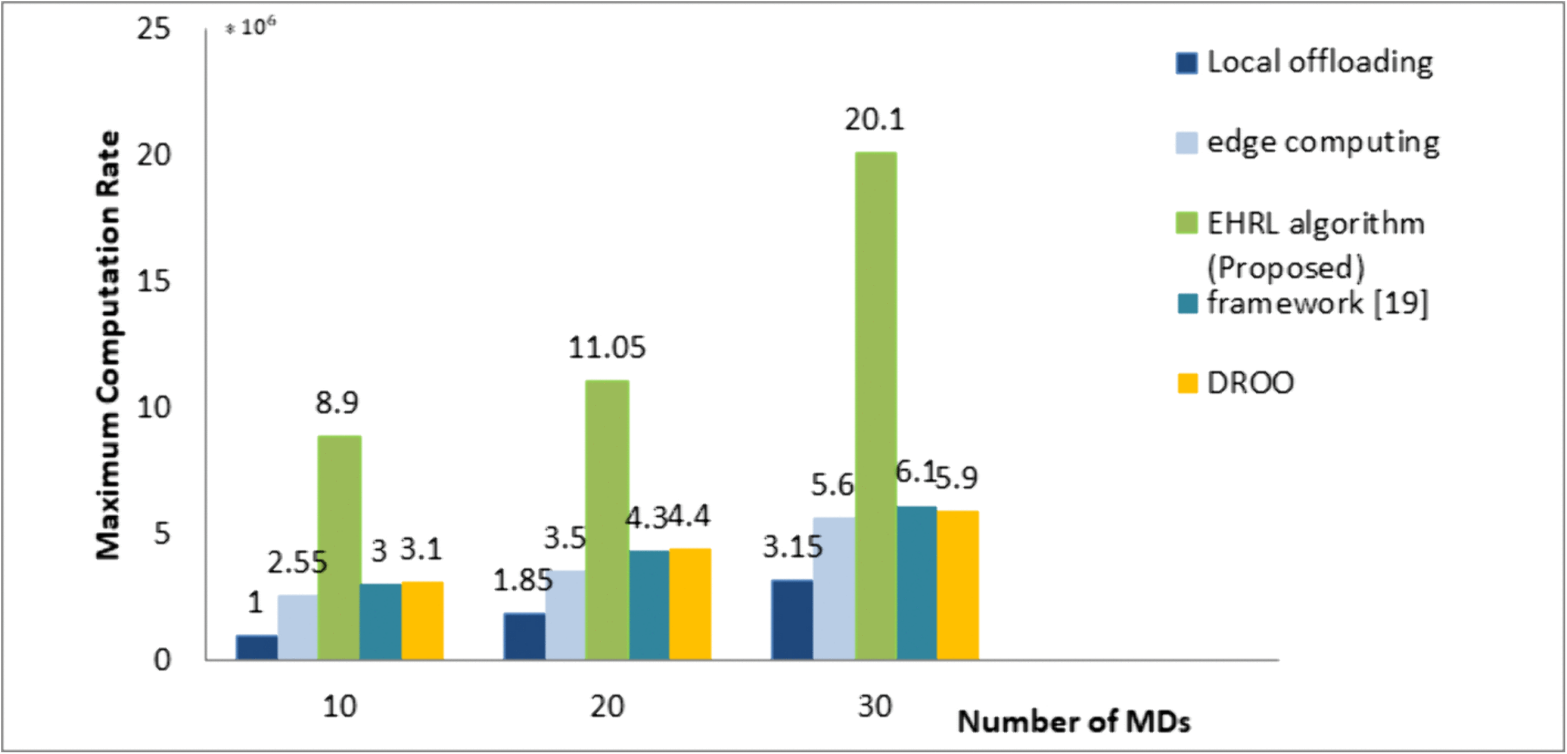
Comparisons of computation rate performance for different offloading algorithms.for N = 10, 20, 30.

To evaluate the computation rate performance, a comparison between local offloading, edge computing, and the algorithm is done under varying numbers of MDs. Fig 7 shows the different values of the maximum computation rate between the different algorithms at N=10, 20, and 30. We see that the algorithm significantly outperforms Edge Computing, Local offloading, and other compared algorithms.

**Fig 8:**
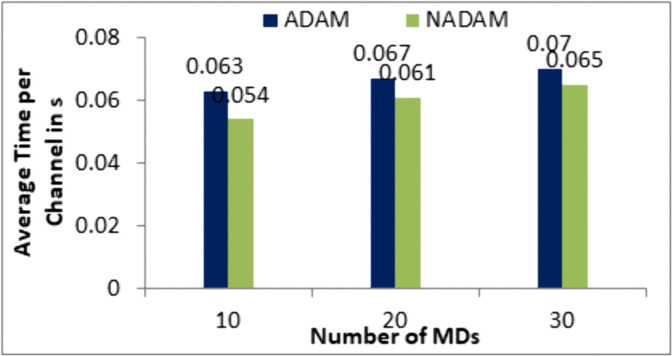
Comparisons between ADAM and NADAM optimizers.

To evaluate the impact of the DNN optimizer on the performance of the proposed work, experiments were conducted using two popular optimizers: NADAM (Nesterov-accelerated Adaptive Moment Estimation) and ADAM (Adaptive Moment Estimation). The results, illustrated in Fig 8, clearly demonstrate that NADAM outperforms ADAM in terms of training convergence speed. This improvement can be attributed to NADAM’s use of Nesterov momentum, which anticipates parameter updates more effectively and leads to faster convergence. These findings validate the choice of NADAM as the optimizer for the DNN, contributing to the overall efficiency and time of the proposed system.

**Fig 9:**
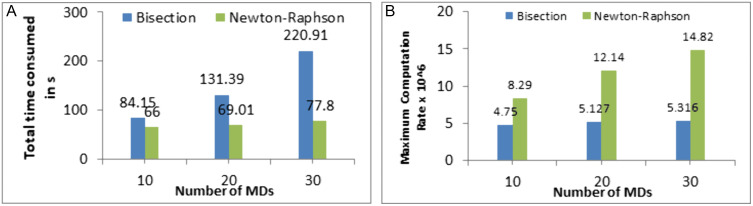
Comparison between Bisection and Newton-Raphson in terms of (A) total time consumed, and (B) maximum computation rate.

In this paper, we adopted the Newton-Raphson method for optimizing the computation rate due to its superior performance compared to the Bisection method. As illustrated in Fig 9A, Fig 9B, Newton-Raphson consistently achieves a higher maximum computation rate and significantly reduces the total time consumed during optimization. Unlike Bisection, which has a linear convergence rate and requires a predefined search interval, Newton-Raphson leverages quadratic convergence to rapidly refine the solution, making it more efficient for solving the non-linear equations in our framework. These advantages validate the use of Newton-Raphson in our work, ensuring a balance between computational efficiency and performance optimization in energy-harvesting MEC systems.

**Table 4:** Performance Comparison of State-of-the-Art WPT-MEC Offloading Methods.

Paper	Model/ Method	Offloading Type	SCR Performance	Execution Latency	Energy Efficiency	Convergence Speed
[12]	Binary offloading optimization (CD & ADMM)	Binary	9.5 (≥ 98%)	Moderate	Moderate	Slow
[13]	DROO (DNN action generator + quantization)	Binary	≥ 99%	Very fast (<0.1s)	High	Fast
[14]	LyDROO (Actor–Critic + model-based critic)	Binary	≈ 100%	Fast	High	Fast
[15]	DRL + Lagrangian dual (time allocation)	Partial	9.5 (≥ 98%)	Fast	High	Fast
[16]	RLIO (CNN-based RL)	Binary	≈ 100%	Ultra-fast (< 1ms)	High	Fast
[17]	Multi-Agent DRL	Binary	9.5 (≥ 98%)	Very fast	High	Fast
[18]	DQN dynamic partial offloading	Binary (multi-AP)	95%	Fast	Moderate	Fast
[19]	Federated Actor–Critic	Binary (OFDMA)	N/A	Moderate (372.3 ms)	Low	Slow
[20]	DRLAO (AugDNN + KOQ + MSM)	Binary	> 98%	Fast	High	Fast
Proposed Work	EHRL (RL–DNN + Newton–Raphson + Nadam)	Binary + Energy Allocation	97–99%	Fast (0.12s)	Very High	Very Fast

A comparative performance analysis was conducted against several state-of-the-art WPT-MEC offloading methods, as summarized in Table 4. The comparison demonstrates that the proposed framework achieves a better balance across multiple performance dimensions. While existing methods such as DROO, LyDROO, and RLIO maintain high Sum Computation Rate (SCR) performance, they often require longer training periods or offer less adaptability in highly dynamic WPT-MEC environments. In contrast, our method not only sustains 97–99% of the optimal SCR with fast decision-making (0.12 s per frame), but also excels in energy efficiency and convergence speed. Specifically, the Newton–Raphson optimization enables rapid convergence and improves energy utilization by reducing computational overhead, leading to the highest energy efficiency among all compared methods.

**Fig 10:**
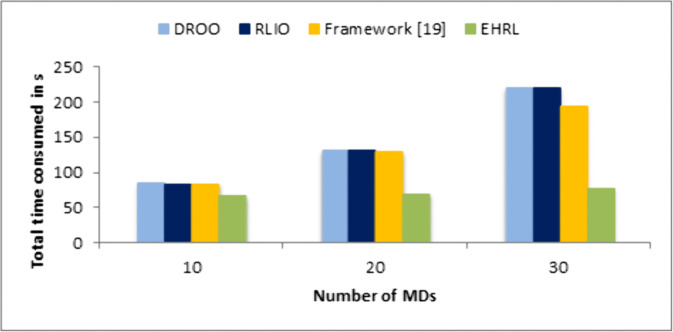
Comparison of total time consumed for different offloading algorithms.for N = 10, 20, 30.

Another comparison is done with other state-of-the-art algorithms in terms of the total time consumed for computation offloading and optimization. The results, illustrated in Fig 10, clearly show that our algorithm outperforms the alternatives by achieving significantly lower total time. This improvement is primarily due to the integration of the Newton-Raphson method, which provides rapid convergence to optimal solutions, unlike the iterative and slower convergence processes of traditional methods. These findings validate the efficiency of our algorithm, making it a superior choice for real-time applications in energy-harvesting MEC systems.

## 6. Conclusion

In this work, an energy-harvesting-enabled computational offloading framework for MEC systems was proposed, leveraging reinforcement learning and the Newton-Raphson method for optimized decision-making. By integrating WPT and energy harvesting, the framework addresses the energy constraints of MDs, enabling sustainable and efficient operation. The DNN was trained using the NADAM optimizer, which outperformed ADAM in terms of convergence speed and stability, ensuring accurate offloading decisions. Furthermore, the Newton-Raphson method proved to be a superior optimization technique, achieving higher computation rates and significantly reducing computational time compared to traditional methods. Simulations validated the framework’s ability to maximize computation rates and minimize total consumed time, showcasing its robustness and adaptability under dynamic network conditions. These results highlight the potential of the proposed framework to enhance the performance of MEC systems. Future work will focus on extending the framework to support diverse offloading scenarios and further improve scalability and adaptability.

## References

[1] Alqarni MM, Cherif A, Alkayal E. A survey of computational offloading in cloud/edge-based architectures: strategies, optimization models and challenges. KSII Transactions on Internet and Information Systems. 2021;15(3).

[2] Zabihi Z, Eftekhari Moghadam AM, Rezvani MH. Reinforcement Learning Methods for Computation Offloading: A Systematic Review. ACM Comput Surv. 2023;56(1):1–41. doi: 10.1145/3603703

[3] Chen X, Liu G. Energy-Efficient Task Offloading and Resource Allocation via Deep Reinforcement Learning for Augmented Reality in Mobile Edge Networks. IEEE Internet Things J. 2021;8(13):10843–56. doi: 10.1109/jiot.2021.3050804

[4] Shi W, Cao J, Zhang Q, Li Y, Xu L. Edge Computing: Vision and Challenges. IEEE Internet Things J. 2016;3(5):637–46. doi: 10.1109/jiot.2016.2579198

[5] Ghosh AM, Grolinger K. Edge-cloud computing for internet of things data analytics: Embedding intelligence in the edge with deep learning. IEEE Transactions on Industrial Informatics. 2021;17(3):2191–200.

[6] Mao Y, You C, Zhang J, Huang K, Letaief KB. A Survey on Mobile Edge Computing: The Communication Perspective. IEEE Commun Surv Tutorials. 2017;19(4):2322–58. doi: 10.1109/comst.2017.2745201

[7] Islam A, Debnath A, Ghose M, Chakraborty S. A Survey on Task Offloading in Multi-access Edge Computing. Journal of Systems Architecture. 2021;118:102225. doi: 10.1016/j.sysarc.2021.102225

[8] Dash SK, Dash S, Mishra J. Opportunistic mobile data offloading using machine learning approach. Wireless Personal Communications. 2020;110:125–39.

[9] Shakya AK, Pillai G, Chakrabarty S. Reinforcement learning algorithms: A brief survey. Expert Systems with Applications. 2023;231:120495. doi: 10.1016/j.eswa.2023.120495

[10] Zhou Z, Chen X, Li E, Zeng L, Luo K, Zhang J. “Edge Intelligence: Paving the Last Mile of Artificial Intelligence With Edge Computing,” in Proceedings of the IEEE, VOL. 107, NO. 8;1738–62, August 2019.

[11] Mao S, Leng S, Maharjan S, Zhang Y. Energy Efficiency and Delay Tradeoff for Wireless Powered Mobile-Edge Computing Systems With Multi-Access Schemes. IEEE Trans Wireless Commun. 2020;19(3):1855–67. doi: 10.1109/twc.2019.2959300

[12] Bi S, Zhang YJ. Computation Rate Maximization for Wireless Powered Mobile-Edge Computing With Binary Computation Offloading. IEEE Trans Wireless Commun. 2018;17(6):4177–90. doi: 10.1109/twc.2018.2821664

[13] Huang L, Bi S, Zhang Y-JA. Deep Reinforcement Learning for Online Computation Offloading in Wireless Powered Mobile-Edge Computing Networks. IEEE Trans on Mobile Comput. 2020;19(11):2581–93. doi: 10.1109/tmc.2019.2928811

[14] Bi S, Huang L, Wang H, Zhang Y-JA. Lyapunov-Guided Deep Reinforcement Learning for Stable Online Computation Offloading in Mobile-Edge Computing Networks. IEEE Trans Wireless Commun. 2021;20(11):7519–37. doi: 10.1109/twc.2021.3085319

[15] Zhang S, Gu H, Chi K, Huang L, Yu K, Mumtaz S. DRL-Based Partial Offloading for Maximizing Sum Computation Rate of Wireless Powered Mobile Edge Computing Network. IEEE Trans Wireless Commun. 2022;21(12):10934–48. doi: 10.1109/twc.2022.3188302

[16] Mustafa E, Shuja J, Bilal K, Mustafa S, Maqsood T, Rehman F, et al. Reinforcement learning for intelligent online computation offloading in wireless powered edge networks. Cluster Comput. 2022;26(2):1053–62. doi: 10.1007/s10586-022-03700-5

[17] Shen G, Chen W, Zhu B, Chi K, Chen X. DRL based binary computation offloading in wireless powered mobile edge computing. IET Communications. 2023;17(15):1837–49. doi: 10.1049/cmu2.12658

[18] Zhang S, Bao S, Chi K, Yu K, Mumtaz S. DRL-Based Computation Rate Maximization for Wireless Powered Multi-AP Edge Computing. IEEE Transactions on Communications. 2024;72(2):1105–18.

[19] Maray M, Mustafa E, Shuja J. Wireless Power Assisted Computation Offloading in Mobile Edge Computing: A Deep Reinforcement Learning Approach. Human-centric Computing and Information Sciences. 2024;14(22).

[20] Wu X, Yan X, Yuan S, Li C. Deep Reinforcement Learning-Based Adaptive Offloading Algorithm for Wireless Power Transfer-Aided Mobile Edge Computing. In: 2024 IEEE Wireless Communications and Networking Conference (WCNC), 2024. 1–6. doi: 10.1109/wcnc57260.2024.10570527

[21] Bi S, Ho CK, Zhang R. Wireless powered communication: opportunities and challenges. IEEE Communications Magazine. 2015;53(4):117–25.

[22] Wang F, Xu J, Wang X, Cui S. Joint Offloading and Computing Optimization in Wireless Powered Mobile-Edge Computing Systems. IEEE Trans Wireless Commun. 2018;17(3):1784–97. doi: 10.1109/twc.2017.2785305

[23] You C, Huang K, Chae H, Kim B-H. Energy-Efficient Resource Allocation for Mobile-Edge Computation Offloading. IEEE Trans Wireless Commun. 2017;16(3):1397–411. doi: 10.1109/twc.2016.2633522

[24] Guo S, Xiao B, Yang Y, Yang Y. Energy-efficient dynamic offloading and resource scheduling in mobile cloud computing. In: Proceedings of IEEE INFOCOM, 2016. 1–9.

[25] You C, Huang K, Chae H. Energy Efficient Mobile Cloud Computing Powered by Wireless Energy Transfer. IEEE J Select Areas Commun. 2016;34(5):1757–71. doi: 10.1109/jsac.2016.2545382

[26] Akram S, Ann Q ul. Newton Raphson Method. International Journal of Scientific & Engineering Research. 2015;6(7).

[27] Dozat T. Incorporating Nesterov Momentum into Adam. In: ICLR Workshop, 2016. 2013–6.

[28] Marsland S. Machine learning: an algorithmic perspective. CRC press. 2015.

[29] Wang Y, Sheng M, Wang X, Wang L, Li J. Partial computation offloading using dynamic voltage scaling. IEEE Transactions on Communications. 2016;64(10):4268–82.

